# Relation Between Humor Styles and Psychopathological Symptoms in Healthcare Professionals: A Cross-Sectional Study

**DOI:** 10.3390/nursrep15010021

**Published:** 2025-01-14

**Authors:** Miriam Leñero-Cirujano, Héctor González-Ordi, Juan Ignacio Torres-González, Jacinto Gómez-Higuera, Sergi Piñar-Rodríguez, Elena López-Bermejo Minaya, Gregorio Jesús Alcalá-Albert, Álvaro Carmona-Pestaña, María Nieves Moro-Tejedor

**Affiliations:** 1 Department of Nursing, Faculty of Medicine, Autonomous University of Madrid, 28029 Madrid, Spain; miriam.lenero@uam.es; 2 Gregorio Marañón Health Research Institute (IiSGM), 28007 Madrid, Spain; mnieves.moro@salud.madrid.org; 3 Faculty of Nursing, Psysiotherapy and Podiatry, Complutense University of Madrid, 28040 Madrid, Spain; hectorgo@psi.ucm.es (H.G.-O.); jacintogomez@enf.ucm.es (J.G.-H.); 4 Transplant Coordination, Clínico San Carlos General University Hospital, 28040 Madrid, Spain; juanignacio.torres@salud.madrid.org; 5 Mental Health Institute of the Hospital del Mar, 08003 Barcelona, Spain; spinar@psmar.cat; 6 Doctoral School, University of Valencia, 46010 Valencia, Spain; lomie@alumni.uv.es; 7 Faculty of Health Sciences, Loyola Andalucía University, 14004 Córdoba, Spain; 8 Faculty of Health Sciences, Loyola Andalucía University, 41704 Seville, Spain; acpestana@uloyola.es; 9 Nursing Research Support Unit, Gregorio Marañón General University Hospital, 28007 Madrid, Spain

**Keywords:** humor, mental health, nursing, nurse, psychopathological symptoms

## Abstract

**Background/Objectives**: Being a healthcare professional often involves exposure to complex situations that can contribute to the development of psychological problems. Evidence suggests that both mental and physical health are crucial for the well-being of these professionals, which in turn influences the quality of care they provide to patients. The main aim of this study was to examine the association between adaptive and maladaptive forms of humor and psychopathological disorders among healthcare workers. **Methods**: A cross-sectional and descriptive correlational design was employed. The participants consisted of 250 healthcare professionals at a general hospital. Humor styles and the presence of psychological and psychosomatic symptoms were assessed. Data were collected using the Humor Styles Questionnaire (HSQ), the Short Checklist of Symptoms (LSB-50), and sociodemographic information. Spearman correlation analysis and linear regression analysis were conducted. **Results**: Healthcare professionals were more inclined to use affiliative and self-enhancing humor styles compared to self-defeating and aggressive humor. Affiliative and self-enhancing humor styles were negatively correlated with psychological symptoms, whereas aggressive humor and, to a lesser extent, self-defeating humor styles showed a significantly positive correlation with most psychological symptoms. Regression analysis revealed that positive humor styles negatively predicted all severity indices of the LSB-50. In contrast, negative humor styles, particularly self-defeating humor, positively predicted the severity indices. Being female, working night shifts, and having temporary contracts were positive predictors of most global indices of the LSB-50. **Conclusions**: This study highlights the importance of considering different types of humor as a potential strategy for improving the mental health of healthcare professionals, as well as the influence of other independent variables related to their personal and work environment. Positive humor styles, specifically affiliative and self-enhancing humor, are associated with a lower prevalence of psychological symptoms among healthcare professionals. Negative humor styles are correlated with a higher prevalence of these symptoms.

## 1. Introduction

Healthcare professions often involve frequent exposure to many adverse and difficult situations that can lead to psychological problems for the healthcare staff [1]. These include prolonged job shifts, work overload, time pressure, demanding work situations, the responsibility of clinical decision-making, exposure to diseases, suffering, and death, the constant need for concentration, and understaffing [2,3].

Chronic exposure to stressors has a potential impact on mental health. Some of the above-mentioned factors have been associated with mental health problems in these professionals, including the risk of developing burnout symptoms and stress-related mental disorders such as depression, anxiety, debilitating sleep disorders, substance misuse, personality disorders, compassion fatigue, reduced job satisfaction [3,4,5], and cardiovascular, digestive, or musculoskeletal disorders [6].

The existence of psychopathology not only causes personal distress but also results in other unfavorable outcomes. Both patients and the organization are adversely affected, which impacts the quality of care and work productivity [7]. Consequently, recognizing those healthcare professionals at risk of developing some kind of psychopathology and identifying help resources constitute important goals [8]. Self-care is not consistently made a priority by healthcare professionals due to concerns about judgment or feeling selfish when considering their own needs. However, prioritizing self-care could be crucial for managing professional obligations, workload, and demands, ultimately facilitating a better balance between work and personal life [8,9]. Thus, it has been suggested that humor is an important mechanism that could improve psychological well-being in healthcare settings [10,11,12].

While a sense of humor is often regarded as a favorable trait, its definition can be ambiguous. Humor encompasses a spectrum of interpretations and involves various cognitive processes and actions [13].

Therapeutic humor, as defined by the Association for Applied and Therapeutic Humor (AATH) is “any intervention that promotes health and wellness by stimulating a playful discovery, expression, or appreciation of the absurdity or incongruity of life’s situations. This intervention may enhance health or be used as a complementary treatment for illness to facilitate healing or coping, whether physical, emotional, cognitive, social, or spiritual” [14].

Several studies have suggested that humor has a beneficial effect on patient care and patient–professional interaction [15], facilitates teamwork, fosters working relationships [16], contributes to job satisfaction and motivation [17], acts as a coping strategy in complicated situations [18], and improves the work environment and the quality of communication [19]. It has been shown that professionals who tend to employ humor more frequently report decreased psychological distress symptoms [18].

Nevertheless, not all forms of humor are beneficial. Specifically, there are two adaptive or positive humor styles: affiliative humor, which facilitates the development of social relationships through telling jokes and humorous stories to amuse others, and self-enhancing humor, which is useful as a coping strategy and helps to find a humorous point of view in stressful situations. Moreover, there are additionally two maladaptive or negative humor styles: aggressive humor, which uses humor to improve one’s own personal image by humiliating others through sarcasm and teasing, and self-defeating humor, which includes excessive behaviors to gain acceptance from others, even at one’s own expense, by employing self-humiliating and self-deprecating jokes. In fact, these humor styles are considered detrimental to oneself and others [20,21]. The use of different humor styles is influenced by social, labor, and cultural factors [22,23]. In a recent study, it was observed that healthcare professionals, in general, scored higher in positive humor styles (affiliative and self-enhancing humor) than in negative humor styles (aggressive and self-defeating humor) [23].

The main aim of this study was to examine the association between adaptive and maladaptive forms of humor and psychopathological disorders among healthcare workers. This study seeks to expand the current understanding of this topic.

The following hypotheses are proposed:

1. Adaptive (or positive) humor styles (affiliative and self-enhancing humor) are associated with a lower presence of psychological symptoms in healthcare workers.
2. Maladaptive (or negative) humor styles (aggressive and self-defeating humor) are associated with a higher presence of psychological symptoms in healthcare workers.

## 2. Materials and Methods

### 2.1. Research Design

This study was conducted using a cross-sectional and descriptive design through the administration of questionnaires. The STROBE checklist for cross-sectional studies was used to report the findings of this study [24].

### 2.2. Sample and Setting

Data were collected from healthcare workers between September and December 2019. Healthcare professionals were recruited at a hospital in Madrid (Spain) using an incidental sampling procedure. The inclusion criteria were being a healthcare professional currently employed at the hospital during the study period. To minimize the risk of biases that could potentially distort the study results and to ensure the reliability of the data obtained, participants who completed less than 80% of the questionnaire were excluded from the analysis. Participation in this study was voluntary and anonymous.

### 2.3. Data Collection

- An informational meeting was held with the supervisors of each unit to clarify this study’s objectives. These supervisors were responsible for distributing the questionnaires to their respective staff members. A total of 800 questionnaires were distributed, each assigned a unique identification number, ensuring the anonymity of the participants. The questionnaires were presented in the following order: Humor Styles Questionnaire; Short Checklist of Symptoms; and sociodemographic data. Permission was obtained from the authors via email to use the scales in this study.
- Humor Styles Questionnaire (HSQ) [21]: this instrument is a self-report questionnaire, structured in 32 items that measure four humor styles (8 items for each humor style): affiliative, self-enhancing, aggressive, and self-defeating. Response options are given on a 7-point Likert scale ranging from 1 (totally disagree) to 7 (totally agree). This structure accounts for 41.60% of the variance and demonstrates a reliability of 0.82. The Spanish version of the HSQ, tested in a sample of healthcare professionals, showed an internal consistency of 0.82 and a common variance of 44.46% [10].
- Short Checklist of Symptoms (LSB-50) [25]: this instrument is a self-report scale comprising 50 items, designed to evaluate different psychological symptoms across seven main clinical scales: hypersensitivity (seven items), which refers to sensitivity to oneself and in relationships with others; obsessive–compulsive (seven items), which covers the presence of doubts, rituals, and compulsions; anxiety (nine items), which enquires about symptoms of panic, general anxiety disorder, and phobic disorders; hostility (six items), which asks about behaviors of rage, anger and resentment; somatization (eight items), which assesses somatic symptoms that are based on psychological or medical problems; depression (ten items), which examines lack of energy and feelings of guilt, sadness, and hopelessness; and sleep disturbance (three items) and extended sleep disturbance (seven items), which enquire about possible sleeping difficulties from a well-being perspective. The severity of symptoms is evaluated through four indices: a global severity index, the number of symptoms, an intensity of symptoms index, and a risk of psychopathology index. Items are rated on a 5-point Likert scale ranging from 0 (nothing) to 4 (a lot). The reliability coefficients for the different scales and indices range from 0.82 to 0.90, and the percentage of explained variance is 55.3%.

### 2.4. Data Analysis

In the data analysis, scores were presented as either means ($\bar{x}$) and standard deviations (SD) or medians (Md) and interquartile ranges ([IQR]), along with minimum (Min) and maximum (Max) ranges in cases where they did not follow a normal distribution. The Spearman correlation coefficient (rho) was examined to assess the association between humor styles and psychopathological disorders. Correlation coefficients between 0.30 and 0.49 were considered a moderate correlation. A linear regression analysis was conducted to determine the predictive power of humor styles and sociodemographic variables across the four indices that assess symptoms severity in the LSB-50: the global severity index, the number of symptoms index, the intensity of symptoms index, and the risk of psychopathology index. Data analysis was conducted using Stata 16.1, with a significance level set at *p* < 0.05 for all tests.

### 2.5. Ethical Considerations

Ethical approval was obtained from the Clinical Research Ethics Committee of the hospital. Participants were provided with an information sheet about this study, along with a consent form, which they were required to sign if they agreed to participate. All participants were adequately informed that their responses would be analyzed as part of a research study. Participation was voluntary, and no compensation was provided. The management of clinical data for this study adhered to the regulations established in Organic Law 3/2018, dated 5 December, concerning the Protection of Personal Data and guarantee of digital rights, as well as the Ethical Standards of the 1964 Declaration of Helsinki.

## 3. Results

A convenience sample of 250 respondents resulted in a 31.25% response rate. Of the total sample, 219 participants were females (87.60%) with ages ranging between 20 and 65 years ($\bar{x}$ = 40.61; SD = 11.40). All participants worked at a hospital in the categories of Registered Nurse (N = 141; 56.40%), nursing assistant (N = 95; 38.00%), doctor (N = 10; 4.00%), and other healthcare worker (N = 4; 1.60%). A detailed overview of the demographic characteristics is presented in Table 1.

Descriptive analyses revealed that the sample exhibited a greater inclination towards utilizing affiliative humor ($\bar{x}$ = 42.45; SD = 7.75) and self-enhancing humor ($\bar{x}$ = 38.23; SD = 8.37) compared to aggressive humor ($\bar{x}$ = 13.36; SD = 5.20) and self-defeating humor ($\bar{x}$ = 22.82; SD = 9.19). Furthermore, our sample scored the highest in the obsessive–compulsive and sleep disturbance clinical scales and the lowest in the hostility and anxiety clinical scales (Table 2).

The analysis of correlations among humor styles and psychopathological symptoms disclosed significant relationships. Negative relationships were observed between affiliative and self-enhancing humor styles and all psychological symptoms and indices (the global severity index, the number of symptoms index, the intensity of symptoms index, and risk of psychopathology index). All correlations were significant, with the exception of the obsessive–compulsive scale and self-enhancing humor (rho = −0.11 [−0.22, −0.02]; *p* = 0.092) (Table 3).

Positive relationships were found between aggressive and self-defeating humor styles with the most psychological symptoms and severity of symptom indices. All correlations were significant in the aggressive humor, with the exception of somatization scale (rho = 0.08 [−0.07, 0.18]; *p* = 0.188), and sleep disturbance scales (rho = 0.12 [0.00, −0.26]; *p* < 0.050). For self-defeating humor, the correlation was only significant with the obsessive–compulsive scale (rho = 0.18 [0.05, 0.28]; *p* = 0.0045), depression scale (rho = 0.13 [0.01, 0.25]; *p* = 0.038, and risk of psychopathology index (rho = 0.14 [0.02, 0.23]; *p* = 0.023) (Table 3).

In the linear regression analysis, the global severity index, the number of symptoms index, the intensity of symptoms index, and the risk of psychopathology index were used as dependent variables, resulting in statistically significant models (*p* < 0.001), with adjusted coefficients of determination ranging from 11.27% to 20.26%. These findings indicate that the predictor variables explain a relevant proportion of the variability in each case.

The model for the global severity index presented an F (11.24) = 6.75, *p* < 0.001, with R^2^ = 0.24 and an adjusted R^2^ of 0.20. Affiliative humor (β = −0.01; *p* = 0.003) and self-enhancing humor (β = −0.02; *p* < 0.001) showed significant negative effects, while self-defeating humor had a positive effect (β = 0.01; *p* = 0.002). Additionally, being female was associated with a significant increase of 0.24 units (*p* = 0.003), and belonging to the “temporary staff” category was related to a significant increase in this variable (β = 0.19; *p* = 0.043). Age and other variables, such as work shift, did not show significant associations (*p* > 0.05) (Table 4).

Similarly, in the number of symptoms index, affiliative humor (β = −0.31; *p* = 0.002) and self-enhancing humor (β = −0.31; *p* < 0.001) had significant negative effects, while self-defeating humor had a positive effect (β = 0.23; *p* = 0.003). Being female was associated with an increase of 5.81 units (*p* = 0.003) in this variable. Employment status as temporary staff (β = 4.44; *p* = 0.045) and night shift work (β = 5.45; *p* = 0.044) also had significant positive effects. The remaining variables did not reach statistical significance (Table 5).

In the case of the intensity of symptoms index, affiliative humor (β = −0.01; *p* = 0.002) and self-enhancing humor (β = −0.01; *p* = 0.000) showed significant negative effects, while self-defeating humor had a positive effect (β = 0.01; *p* = 0.003). Being female (β = 0.12; *p* = 0.003), employment status as temporary staff (β = 0.09; *p* = 0.045), and night shift work (β = 0.12; *p* = 0.044) also had significant positive effects on the variable (Table 6).

Finally, in the risk of psychopathology index, affiliative humor (β = −0.01; *p* = 0.016) and self-enhancing humor (β = −0.01; *p* = 0.002) showed significant negative effects, while self-defeating humor had a positive effect (β = 0.01; *p* = 0.002). Being female (β = 0.16; *p* = 0.032) and employment status of temporary staff (β = 0.18; *p* = 0.032) also had significant positive effects on the variable (Table 7).

## 4. Discussion

This study explored how adaptive and maladaptive humor styles relate to psychopathological symptoms among healthcare professionals. Healthcare professionals were more inclined to use affiliative and self-enhancing humor styles compared to self-defeating and aggressive humor, suggesting a predominance of positive humor styles among healthcare professionals [23].

Overall, the literature indicates that humor can enhance well-being, although its effects vary depending on different styles of humor [26,27,28]. In fact, this study shows that positive humor styles—affiliative and self-enhancing humor—are moderately, negatively, and significantly associated with most of the LSB-50 scales and indices of severity, intensity, and number of symptoms. Additionally, the regression models indicated that positive humor negatively predicted all severity indices of the LSB-50. These findings align with previous studies indicating that a propensity for employing benign humor styles is correlated with heightened well-being in healthcare environments. The negative association between adaptive humor styles and other negative psychological variables such as anxiety [26], depression [11], or sadness has been extensively investigated [29]. Other investigations have also suggested that affiliative and self-enhancing humor styles correlated positively with extroversion, personal attraction and cohesion, self-esteem, optimism, satisfaction with relationships, and other positive variables [12,27,28,30].

Concerning the utilization of aggressive humor, there was a weak and negative association with LSB-50 scales and indexes, although this type of humor did not significantly predict any of them in the regression models. In the literature, a propensity for aggressive humor has been shown to correlate with poorer psychological functioning [12]. Scientific studies have shown a positive correlation between aggressive humor and aggressiveness, hostility, neuroticism, anxiety, paranoid ideation, and the global severity index [20,27,31], as well as a negative correlation between optimism, satisfaction with relationships with colleagues at work and well-being [27,32]. However, other studies have documented a lack of substantial correlation between aggressive humor and psychological well-being [33,34]. Therefore, future research should explore whether the relationship between aggressive humor and specific scales and indices of LSB-50 might vary across different contexts.

Ultimately, self-defeating humor was positively correlated with most of the LSB-50 indices and scales. However, this type of humor has been negatively but not significantly associated with somatization scale. Additionally, in the regression models, self-defeating humor positively and significantly predicted severity, intensity, and number of symptoms. This finding may help explain the dual role that this type of humor plays, depending on whether its use is intentional or unintentional. When employed as a resource for self-acceptance, as an act of personal acknowledgment, it could aid in the regulation of emotional control, such as aggression, anger, or rage, as well as other somatic disorders. Furthermore, it has been suggested that the humor found in laughing at oneself may be linked to prosocial behaviors associated with agreeableness, self-esteem, and extroversion [10,12,35]. On the other hand, if used in a self-deprecating manner, it could have a negative impact on psychological well-being. These findings are consistent with the results described in other studies, where positive and significant correlations were found between this type of humor and indices of paranoid ideation, psychoticism, and global symptom severity, among others [20,31]. This humor has been directly associated with neuroticism, hostility, anxiety, depression, lack of well-being, defensive denial, shyness, and other psychiatric symptoms in other studies [11,36,37,38,39].

In summary, based on these findings, it could be affirmed that affiliative and self-enhancing humor is linked to the psychological well-being of healthcare professionals, while aggressive and self-defeating humor is associated with a higher presence of psychopathological symptoms. However, healthcare professionals exhibit different humor styles influenced by their social and work environments [23]. As explained in the regression models, the LSB-50 indices are also explained by other independent variables that need to be considered. Considering the regression models in this study, the following factors are positive predictors of the global severity indices of the LSB-50: being a woman, working night shifts, and having a temporary contract. These findings align with those described in other studies, which indicate higher rates of psychopathological disorders in these populations [23,25,40,41,42,43]. Additional research is needed to ascertain whether these differences are explained by contextual factors and cultural or occupational aspects among healthcare professionals.

It should be noted that our study has limitations. Firstly, the sample size was relatively small, largely owing to difficulties in accessing this specific population. Although a high response rate is always desirable, achieving higher rates in studies involving healthcare professionals is often challenging. The response rate obtained is comparable to that of other studies conducted with samples of healthcare professionals [44]. While we believe there was some level of motivation among the participants, it is important to consider potential factors contributing to non-responses when interpreting the results, such as the length of the administered questionnaires, time constraints, workload, and lack of motivation, among others. The incidental sampling procedure and non-experimental approach employed in our study preclude definitive causal inferences from its results. However, the consistent findings regarding the positive effects of humor on psychological well-being instill confidence that our results may be replicable in larger samples. Future research should employ random sampling methods and longitudinal designs, as well as further explore the direction of the relationships between humor styles and psychosomatic symptoms identified in this study.

The results of our study make a significant contribution to the recognition and awareness of the importance of identifying measures that address the psychological needs of healthcare professionals and promote their well-being. The recommendations derived from this article for practical implementation include training professionals in the use of positive humor, as well as the implementation of workplace programs aimed at fostering this humor style. These measures aim to create a healthier work environment and mitigate the impact of negative humor styles that could hinder the well-being of healthcare professionals.

## 5. Conclusions

This study highlights the importance of considering different types of humor as a potential strategy for improving the mental health of healthcare professionals, as well as the influence of other independent variables related to their personal and work environment. It finds that positive humor styles, specifically affiliative and self-enhancing humor, are associated with a lower prevalence of psychological symptoms among healthcare professionals and negatively predict all severity indices of the LSB-50. In contrast, negative humor styles, particularly aggressive humor, are correlated with a higher prevalence, severity, and intensity of symptoms such as hypersensitivity, obsessive–compulsive behavior, anxiety, hostility, depression, and risk of psychopathology. Self-defeating humor is correlated with a higher prevalence of symptoms such as obsessive–compulsive behavior, depression, and risk of psychopathology, with the latter positively predicting the severity indices of the LSB-50.

## Figures and Tables

**Table 1:** Demographic and labor characteristics of participants (N = 250).

Variables	N (%)
**Gender**	
Female	219 (87.60%)
Male	31 (12.40%)
**Marital status**	
Married	111 (44.40%)
Divorcee	21 (8.40%)
Single	115 (46.00%)
Widow	3 (1.20%)
**Professional category**	
Doctor	10 (4.00%)
Nurse	141 (56.40%)
Nursing Assistant	95 (38.00%)
Others	4 (1.60%)
**Shift work**	
Morning	140 (56.00%)
Afternoon	75 (30.00%)
Night	15 (6.00%)
Rotating	20 (8.00%)
**Type of contract**	
Permanent	95 (38.00%)
Interim	110 (44.00%)
Temporary	41 (16.40%)
Training	4 (1.60%)

**Table 2:** Descriptive statistics of the Humor Styles Questionnaire and the Short Checklist of Symptoms (N = 250).

Scales	$\bar{x}$	SD	Md	IQR	Min	Max
**Humor Styles Questionnaire**						
Affiliative	42.45	7.75	43.00	[37.00–48.00]	12	56
Self-enhancing	38.23	8.37	39.50	[33.00–44.25]	8	36
Aggressive	13.36	5.20	12.00	[9.00–16.00]	8	50
Self-defeating	22.82	9.19	22.00	[16.00–29.00]	55	181
**Short Checklist of Symptoms**						
Hypersensitivity	0.52	0.56	0.43	[0.15–0.71]	0	2.86
Obsessive–compulsive	0.86	0.52	0.86	[0.43–1.14]	0	2.57
Anxiety	0.36	0.49	0.22	[0.00–0.44]	0	3.00
Hostility	0.42	0.47	0.33	[0.00–0.54]	0	2.33
Somatization	0.68	0.64	0.50	[0.22–1.13]	0	3.25
Depression	0.59	0.57	0.40	[0.20–0.90]	0	2.60
Sleep disturbance	0.93	1.04	0.67	[0.00–1.67]	0	4.00
Extended sleep disturbance	0.69	0.70	0.43	[0.14–1.00]	0	3.29
Global severity	0.59	0.45	0.48	[0.24–0.84]	0	2.06
Number of symptoms	16.80	10.77	15.00	[7.75–25.25]	0	44.00
Intensity of symptoms	0.34	0.22	0.30	[0.16–0.51]	0	0.88
Risk of psychopathology	0.26	0.38	0.08	[0.00–0.33]	0	1.92

**Table 3:** Correlations among humor styles and psychopathological symptoms (N = 250).

		Affiliative	Self- Enhancing	Aggressive	Self- Defeating
Hypersensitivity	rho CI 95% *p*-value	−0.34 ** [−0.48, −0.19] 0.000	−0.25 ** [−0.39, −0.09] 0.000	0.17 ** [0.05, 0.36] 0.006	0.12 [−0.01, 0.24] 0.066
Obsessive–Compulsive	rho CI 95% *p*-value	−0.27 ** [−0.40, −0.10] 0.000	−0.11 [−0.22, −0.02] 0.092	0.13 * [0.03, 0.26] 0.038	0.18 * [0.05, 0.28] 0.045
Anxiety	rho CI 95% *p*-value	−0.18 ** [−0.35, −0.03] 0.004	−0.20 ** [−0.34. −0.03] 0.002	0.14 * [0.04, 0.27] 0.029	0.09 [−0.08, 0.17] 0.119
Hostility	rho CI 95% *p*-value	−0.24 ** [−0.37, −0.06] 0.000	−0.20 ** [−0.39, −0.08] 0.002	0.22 ** [0.04, 0.35] 0.001	0.02 [−0.16, 0.08] 0.760
Somatization	rho CI 95% *p*-value	−0.16 * [−0.24, −0.03] 0.013	−0.28 ** [−0.42, −0.12] 0.000	0.08 [−0.07, 0.18] 0.188	−0.02 [−0.14, 0.11] 0.700
Depression	rho CI 95% *p*-value	−0.27 ** [−0.41, −0.11] 0.000	−0.29 ** [−0.43, −0.14] 0.000	0.15 * [0.01, 0.32] 0.022	0.13 * [0.01, 0.25] 0.038
Sleep disturbance	rho CI 95% *p*-value	−0.25 ** [−0.41, −0.11] 0.000	−0.24 ** [−0.4, −0.09] 0.000	0.12 [0.00, 0.26] 0.057	0.12 [−0.05, 0.20] 0.062
Extended sleep disturbance	rho CI 95% *p*-value	−0.28 ** [−0.41, −0.11] 0.000	−0.30 ** [−0.43, −0.13] 0.000	0.12 [0.00, 0.26] 0.054	0.11 [−0.02, 0.22] 0.096
Global severity	rho CI 95% *p*-value	−0.30 ** [−0.44, −0.14] 0.000	−0.29 ** [−0.44, −0.14] 0.000	0.16* [0.00–0.32] 0.013	0.11 [−0.05, 0.20] 0.098
Number of symptoms	rho CI 95% *p*-value	−0.30 ** [−0.44, −0.15] 0.000	−0.27 ** [−0.41, −0.11] 0.000	0.15 * [0.02–0.26] 0.015	0.11 [−0.05, 0.19] 0.099
Intensity of symptoms	rho CI 95% *p*-value	−0.30 ** [−0.44, −0.15] 0.000	−0.27 ** [−0.41, −0.11] 0.000	0.15 * [0.02–0.26] 0.015	0.11 [−0.05, 0.19] 0.099
Risk of psychopathology	rho CI 95% *p*-value	−0.20 ** [−0.38, −0.07] 0.001	−0.21 ** [−0.36, −0.05] 0.001	0.13 * [0.01, 0.26] 0.043	0.14 * [0.02, 0.23] 0.024

*. Correlation is significant at the 0.05 level (two-tailed). **. Correlation is significant at the 0.01 level (two-tailed).

**Table 4:** Linear regression model for predicting the global severity index (N = 250).

Variable: Global Severity Index	β	SE	95% CI		t	*p* (Sig.)
			Lower	Upper		
Constant	1.03	0.29	0.47	1.59	3.61	0.000
Affiliative humor	−0.01	0.00	−0.02	−0.00	−2.98	0.003
Self-enhancing humor	−0.02	0.00	−0.02	−0.01	−4.31	0.000
Aggressive humor	0.01	0.01	−0.00	0.02	1.62	0.107
Self-defeating humor	0.01	0.00	0.00	0.02	3.12	0.002
Age	0.00	0.00	−0.01	0.01	0.23	0.820
Gender (female)	0.24	0.08	0.08	0.39	3.00	0.003
Type of contract (temporary staff)	0.19	0.09	0.01	0.37	2.04	0.043
Type of contract (interim staff)	0.11	0.06	−0.02	0.23	1.63	0.105
Work shift (night shift)	0.16	0.11	−0.06	0.37	1.44	0.152
Work shift (rotating shift)	0.08	0.10	−0.12	0.28	0.83	0.409
Work shift (afternoon shift)	−0.09	0.06	−0.21	0.31	−1.47	0.144

Note: F (11. 24) = 6.75; *p* < 0.001; R^2^ = 0.24; adjusted R^2^ = 0.20; Root MSE = 0.40. CI = confidence intervals; β = regression coefficient; SE: standard error.

**Table 5:** Linear regression model for predicting the number of symptoms index (N = 250).

Variable: Number of Symptoms Index	β	SE	95% CI		t	*p* (Sig.)
			Lower	Upper		
Constant	26.93	6.87	13.39	40.47	3.92	0.000
Affiliative humor	−0.31	0.10	−0.49	−0.12	−3.21	0.002
Self-enhancing humor	−0.31	0.09	−0.47	−0.14	−3.60	0.000
Aggressive humor	0.15	0.13	−0.10	0.40	1.17	0.245
Self-defeating humor	0.23	0.08	0.08	0.38	3.03	0.003
Age	0.02	0.08	−0.13	0.17	0.27	0.790
Gender (female)	5.81	1.91	2.04	9.58	3.04	0.003
Type of contract (temporary staff)	4.44	2.20	0.09	8.78	2.01	0.045
Type of contract (interim staff)	2.28	1.56	−0.79	5.36	1.46	0.145
Work shift (night shift)	5.45	2.68	0.16	10.73	2.03	0.044
Work shift (rotating shift)	−0.01	2.44	−4.82	4.80	−1.01	0.996
Work shift (afternoon shift)	−2.24	1.48	−5.15	0.67	−1.51	0.131

Note: F (11. 24) = 6.23, *p* < 0.001; R^2^ = 0.22; adjusted R^2^ = 0.19; Root MSE = 9.70. CI = confidence intervals; β = regression coefficient; SE: standard error.

**Table 6:** Linear regression model for predicting the intensity of symptoms index (N = 250).

Variable: Intensity of Symptoms Index	β	SE	95% CI		t	*p* (Sig.)
			Lower	Upper		
Constant	0.54	0.14	2.27	0.81	3.92	0.000
Affiliative humor	−0.01	0.00	−0.01	−0.00	−3.21	0.002
Self-enhancing humor	−0.01	0.00	−0.01	−0.00	−3.60	0.000
Aggressive humor	0.00	0.00	−0.00	0.01	1.17	0.245
Self-defeating humor	0.01	0.00	0.00	0.01	3.03	0.003
Age	0.00	0.00	−0.00	0.00	0.27	0.790
Gender (female)	0.12	0.04	0.04	0.19	3.04	0.003
Type of contract (temporary staff)	0.09	0.04	0.00	0.18	2.01	0.045
Type of contract (interim staff)	0.05	0.03	−0.02	0.11	1.46	0.145
Work shift (night shift)	0.12	0.05	0.00	0.21	2.03	0.044
Work shift (rotating shift)	−0.00	0.05	−0.10	0.10	−0.01	0.996
Work shift (afternoon shift)	−0.04	0.03	−0.10	0.01	−1.51	0.131

Note: F (11. 24) = 6.23, *p* < 0.001; R^2^ = 0.22; adjusted R^2^ = 0.19; Root MSE = 0.19. CI = confidence intervals; β = regression coefficient; SE: standard error.

**Table 7:** Linear regression model for predicting the risk of psychopathology index (N = 250).

Variable: Risk of Psychopathology Index	β	SE	95% CI		t	*p* (Sig.)
			Lower	Upper		
Constant	0.45	0.26	−0.05	0.96	1.76	0.080
Affiliative humor	−0.01	0.00	−0.02	−0.00	−2.42	0.016
Self-enhancing humor	−0.01	0.00	−0.02	−0.00	−3.06	0.002
Aggressive humor	0.00	0.00	−0.01	0.01	0.77	0.441
Self-defeating humor	0.01	0.00	0.00	0.01	3.07	0.002
Age	0.00	0.00	−0.00	0.01	0.71	0.477
Gender (female)	0.16	0.07	0.01	0.29	2.16	0.032
Type of contract (temporary staff)	0.18	0.08	0.02	0.34	2.16	0.032
Type of contract (interim staff)	0.08	0.06	−0.03	0.19	1.38	0.170
Work shift (night shift)	0.16	0.10	−0.04	0.36	1.60	0.110
Work shift (rotating shift)	0.08	0.09	−0.10	0.26	0.86	0.389
Work shift (afternoon shift)	−0.00	0.06	−0.11	0.10	−0.07	0.942

Note: F (11. 24) = 3.88, *p* < 0.001; R^2^ = 0.15; adjusted R^2^ = 0.11; Root MSE = 0.36. CI = confidence intervals; β = regression coefficient; SE: standard error.

## Data Availability

The raw data supporting the conclusions of this article will be made available by the authors on request.

## Public Involvement Statement

Participation was voluntary, and no compensation was provided.

## Guidelines and Standards Statement

This manuscript was drafted against the STROBE checklist for cross-sectional research.

## References

[1] Woo T., Ho R., Tang A., Tam W. (2020). Global prevalence of burnout symptoms among nurses: A systematic review and meta-analysis. J. Psychiatr. Res..

[2] Molina-Praena J., Ramirez-Baena L., Gómez-Urquiza J.L., Cañadas G.R., De la Fuente E.I., Cañadas-De la Fuente G.A. (2018). Levels of Burnout and Risk Factors in Medical Area Nurses: A Meta-Analytic Study. Int. J. Environ. Res. Public Health..

[3] Sullivan D., White K.M., Frazer C. (2022). Factors Associated with Burnout in the United States Versus International Nurses. Nurs. Clin. N. Am..

[4] Varghese A., George G., Kondaguli S.V., Naser A.Y., Khakha D.C., Chatterji R. (2021). Decline in the mental health of nurses across the globe during COVID-19: A systematic review and meta-analysis. J. Glob. Health.

[5] Zhang Y.-Y., Han W.-L., Qin W., Yin H.-X., Zhang C.-F., Kong C., Wang Y.-L. (2018). Extent of compassion satisfaction, compassion fatigue and burnout in nursing: A meta-analysis. J. Nurs. Manag..

[6] Bazazan A., Dianat I., Bahrampour S., Talebian A., Zandi H., Sharafkhaneh A., Maleki-Ghahfarokhi A. (2019). Association of musculoskeletal disorders and workload with work schedule and job satisfaction among emergency nurses. Int. Emerg. Nurs..

[7] Jun J., Ojemeni M.M., Kalamani R., Tong J., Crecelius M.L. (2021). Relationship between nurse burnout, patient and organizational outcomes: Systematic review. Int. J. Nurs. Stud..

[8] Santolalla-Arnedo I., Pozo-Herce P.D., Gea-Caballero V., Juarez-Vela R., Gil-Fernandez G., Garrido-Garcia R., Echaniz-Serrano E., Czapla M., Rodriguez-Velasco F.J. (2022). Psychological impact on care professionals due to the SARS-Cov-2 virus in Spain. Int. Nurs. Rev..

[9] Søvold L.E., Naslund J.A., Kousoulis A.A., Saxena S., Qoronfleh M.W., Grobler C., Münter L. (2021). Prioritizing the Mental Health and Well-Being of Healthcare Workers: An Urgent Global Public Health Priority. Front. Public Health.

[10] Leñero-Cirujano M., Torres-González J.I., González-Ordi H., Gómez-Higuera J., Moro-Tejedor M.N. (2022). Validation of the humour styles questionnaire in healthcare professionals. Nurs. Open.

[11] Menéndez-Aller A., Postigo A., Montes-Álvarez P., González-Primo F.J., García-Cueto E. (2020). Humor as a protective factor against anxiety and depression. Int. J. Clin. Health Psychol..

[12] Navarro-Carrillo G., Torres-Marín J., Corbacho-Lobato J.M., Carretero-Dios H. (2020). The effect of humour on nursing professionals’ psychological well-being goes beyond the influence of empathy: A cross-sectional study. Scand. J. Caring Sci..

[13] Martin R.A., Gallagher M.W., Lopez S.J. (2019). Humor. Positive Psychological Assessment: A Handbook of Models and Measures.

[14] (2015). Association for Applied and Therapeutic Humor. Association for Applied and Therapeutic Humor. https://www.aath.org.

[15] Sousa L., Marques-Vieira C., Antunes A.V., Frade M., Severino S., Valentim O.S. (2019). Humor intervention in the nurse-patient interaction. Rev. Bras. Enferm..

[16] Bartzik M., Bentrup A., Hill S., Bley M., von Hirschhausen E., Krause G., Ahaus P., Dahl-Dichmann A., Peifer C. (2021). Care for Joy: Evaluation of a Humor Intervention and Its Effects on Stress, Flow Experience, Work Enjoyment, and Meaningfulness of Work. Front. Public Health.

[17] Robert C., Da Motta S.P. (2017). Conversational humor and job satisfaction at work: Exploring the role of humor production, appreciation, and positive affect. Humor.

[18] Robalo Nunes I., José H., Capelas M.L. (2018). Grieving with Humor: A Correlational Study on Sense of Humor and Professional Grief in Palliative Care Nurses. Holist. Nurs. Pract..

[19] Brender-Ilan Y., Reizer A. (2021). How Do We Perceive a Humorous Manager? Manager Humor, Impression Management, and Employee Willingness to Work with the Manager. Front. Psychol..

[20] Chen G.H., Martin R.A. (2007). A comparison of humor styles, coping humor, and mental health between Chinese and Canadian university students. Humor.

[21] Martin R.A., Puhlik-Doris P., Larsen G., Gray J., Weir K. (2003). Individual differences in uses of humor and their relation to psychological well-being: Development of the Humor Styles Questionnaire. J. Res. Pers..

[22] Jiang T., Li H., Hou Y. (2019). Cultural Differences in Humor Perception, Usage, and Implications. Front. Psychol..

[23] Leñero-Cirujano M., Moro-Tejedor M.N., Torres-González J.I., González-Ordi H., Gómez-Higuera J. (2024). Humor Styles in Healthcare Professionals: A Correlational Study. Holist. Nurs. Pract..

[24] STROBE STROBE Checklists [Internet]. https://www.strobe-statement.org/checklists/.

[25] Abuín M.R., de Rivera L. (2014). The measurement of psychological and psychosomatic symptoms: The Brief Symptom Inventory (LSB-50). Clin. Salud.

[26] Demir M. (2020). The Effect of Laughter Therapy on Anxiety: A Meta-analysis. Holist. Nurs. Pract..

[27] Jiang F., Lu S., Jiang T., Jia H. (2020). Does the Relation Between Humor Styles and Subjective Well-Being Vary Across Culture and Age? A Meta-Analysis. Front. Psychol..

[28] Schneider M., Voracek M., Tran U.S. (2018). “A joke a day keeps the doctor away?” Meta-analytical evidence of differential associations of habitual humor styles with mental health. Scand. J. Psychol..

[29] Zhao J., Yin H., Zhang G., Li G., Shang B., Wang C., Chen L. (2019). A meta-analysis of randomized controlled trials of laughter and humour interventions on depression, anxiety and sleep quality in adults. J. Adv. Nurs..

[30] Plessen C.Y., Franken F.R., Ster C., Schmid R.R., Wolfmayr C., Mayer A.-M., Sobisch M., Kathofer M., Rattner K., Kotlyar E. (2020). Humor styles and personality: A systematic review and meta-analysis on the relations between humor styles and the Big Five personality traits. Personal. Individ. Differ..

[31] Brizzio A., Carreras C., Casullo M. (2006). Humor and Psychopathological Symptoms: A Study with Argentine Adolescents and Young Adults. Investig. Psicológicas.

[32] Chen H., Ayoun B. (2019). Is negative workplace humor really all that “negative”? Workplace humor and hospitality employees’ job embeddedness. Int. J. Hosp. Manag..

[33] Ruch W., Heintz S. (2013). Humour styles, personality and psychological well-being: What’s humour got to do with it?. Eur. J. Humour Res..

[34] Taher D., Kazarian S.S., Martin R.A. (2008). Validation of the arabic humor styles questionnaire in a community sample of lebanese in lebanon. J. Cross Cult. Psychol..

[35] Ruch W., Heintz S. (2017). Experimentally Manipulating Items Informs on the (Limited) Construct and Criterion Validity of the Humor Styles Questionnaire. Front. Psychol..

[36] Bressington D., Mui J., Yu C., Leung S.F., Cheung K., Wu C.S.T., Bollard M., Chien W.T. (2019). Feasibility of a group-based laughter yoga intervention as an adjunctive treatment for residual symptoms of depression, anxiety and stress in people with depression. J. Affect. Disord..

[37] Erickson S.J., Feldstein S.W. (2007). Adolescent humor and its relationship to coping, defense strategies, psychological distress, and well-being. Child Psychiatry Hum. Dev..

[38] Maiolino N.B., Kuiper N.A. (2014). Integrating Humor and Positive Psychology Approaches to Psychological Well-Being. Eur. J. Psychol..

[39] Saraglou V., Scariot C. (2002). Humor Styles Questionnaire: Personality and Educational Correlates in Belgian High School and College Students. Eur. J. Pers..

[40] Albendína L., Gómez J.L., Cañadas-de la Fuente G.A., Cañadas G.R., San Luis C., Aguayo R. (2016). Prevalencia bayesiana y niveles de burnout en enfermería de urgencias. Una revisión sistemática. Rev. Latinoam. Psicol..

[41] Jørgensen J.T., Rozing M.P., Westendorp R.G.J., Hansen J., Stayner L.T., Simonsen M.K., Andersen Z.J. (2021). Shift work and incidence of psychiatric disorders: The Danish nurse cohort study. J. Psychiatr. Res..

[42] Torquati L., Mielke G.I., Brown W.J., Burton N.W., Kolbe-Alexander T.L. (2019). Shift Work and Poor Mental Health: A Meta-Analysis of Longitudinal Studies. Am. J. Public Health.

[43] Rubinow D.R., Schmidt P.J. (2019). Sex differences and the neurobiology of affective disorders. Neuropsychopharmacology.

[44] Fang C., Fan S., Chen D., Zhou Y., Fan W. (2024). The relation between humor styles and nurse burnout: A cross-sectional study in China. Front. Public Health.

